# Gender Differences in the Cortical Distribution of Corpus Callosum Fibers

**DOI:** 10.7759/cureus.55918

**Published:** 2024-03-10

**Authors:** Mudathir Bakhit, Masazumi Fujii

**Affiliations:** 1 Neurosurgery, Fukushima Medical University, Fukushima, JPN

**Keywords:** white matter tracts, tractography, sex-based differences, corpus callosum, commissural fibers

## Abstract

Introduction

Research on gender-based disparities in human brain structure has spanned over a century, yielding conflicting results and ongoing debate. While some studies indicate minimal distinctions, others consistently highlight differences in the corpus callosum (CC), even after accounting for average brain size.

Methods

Diverging from previous approaches, this study examines the morphology of the entire CC fiber rather than solely focusing on its midsagittal structure. Utilizing advanced neuroimaging techniques and generalized Q-imaging tractography, CC streamlines were constructed to assess gender differences in fractional anisotropy (FA), volume ratio, and cortical distribution. Student’s t-test was employed to examine the disparities in FA between gender groups, while gender-based distinctions in the normalized volume of the CC and its segments were assessed using analysis of covariance (ANCOVA), with absolute whole white matter volume serving as a covariate.

Results

No significant gender-based disparities were found in either FA or normalized CC volume. While females exhibited consistently larger normalized volume CC streamlines than males, these differences lost statistical significance after adjusting for absolute total white matter volume as a covariate. Nonetheless, CC streamlines in females displayed a broader spatial distribution, encompassing various cortical regions, including the bilateral prefrontal cortex (medial and lateral surfaces), as well as medial parietal and temporal regions.

Conclusion

This study elucidates gender-related variations in the morphology of the brain's white matter pathways, indicating a more widespread cortical distribution of CC fibers in females compared to males. However, the study underscores the need for further investigations into connectivity patterns to fully elucidate these gender-based disparities.

## Introduction

The debate over gender differences in the human brain is fueled by contradictory study findings, some suggesting minimal differences while others highlighting reproducible disparities, even after adjusting for the average brain size [1]. A recent study analyzed over 2,000 brain scans and showed reproducible gender-based differences in brain volume. Moreover, they found a significant association between these differences and the expression of sex chromosome genes [2]. Diffusion-weighted magnetic resonance imaging (MRI) and tractography revealed sex-specific differences in white matter pathways linked to verbal abilities, including distinct fractional anisotropy (FA) and radial diffusivity patterns between males and females [3]. A separate study examining gender differences in white matter structure revealed volumetric asymmetry in specific association white matter tracts between males and females [4].

In the domain of white matter gender-based differences, the corpus callosum (CC) has been one of the most extensively studied white matter bundles. The works of Holloway et al. and De Lacoste-Utamsing et al. were among the early in this field [5,6]. They found that the splenial segment of the CC was more bulbous in females’ brains and that the absolute and relative values of the total area of the CC were higher in females. More recently, Kanaan et al. reported gender differences in white matter microstructure, with women showing increased FA in the CC [7]. Shiino et al. found that the CC volume was larger in females compared to males in 37 total intracranial volume-matched pairs sample [8]. Using the same sample, Ardekani et al. investigated the differences in the CC area and found it significantly larger in females [9]. However, studies that found no significant gender-based differences are present. Luders et al. found no callosal differences between brain-size-matched males and females [10].

In the current study, we focused on the morphology of the fibers passing through the CC and not the midsagittal structure. Using advanced neuroimaging techniques and high-quality brain imaging data, we applied generalized Q-imaging (GQI) tractography [11] to construct CC streamlines. These streamlines were then utilized to evaluate their FA and volume ratio in relation to the total white matter volume (normalized volume). We also studied the anatomico-spatial distribution of these fibers in both hemispheres and outlined any gender differences that can be detected.

A copy of this article was orally presented (in Japanese) at the 17th Japan Association for Gender-Specific Medicine Meeting (January 27-28, 2024).

## Materials and methods

Dataset

**Figure 1:**
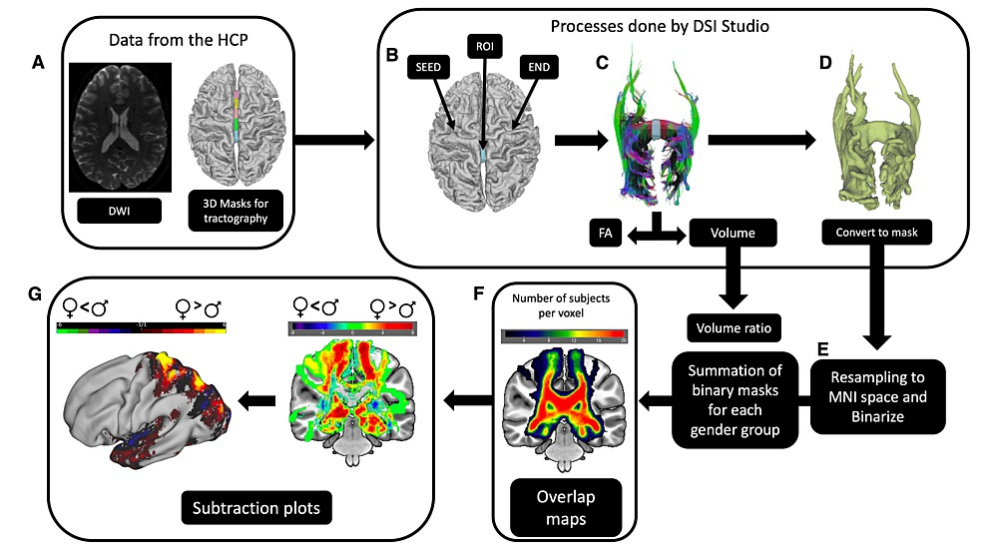
Overview of the study methodology. (A) Neuroimaging data, including diffusion-weighted images (DWI) and 3D volumetric masks from Freesurfer's atlas, were sourced from the Human Connectome Project (HCP) database. These data were utilized for GQI- tractography using DSI Studio software. (B) Within each subject, the left hemisphere white matter mask served as a seed, the right hemisphere white matter mask as an end, and one of the five segments of the corpus callosum (CC) as the region of interest (ROI). (C) Tractography results underwent manual confirmation of target streamlines, followed by the extraction of volume and fractional anisotropy values. (D) The selected streamlines were transformed into 3D volumetric masks. (E) Subsequently, these masks were resampled to the Montreal Neurological Institute (MNI) standard space and binarized. (F) Masks from corresponding CC segments within each gender group were summed to create overlap maps. (G) The CC segment with the smaller mean volume was subtracted from the one with the larger mean volume to generate a subtraction plot. This plot was then visualized and overlaid on a 3D brain surface to explore cortical surface distribution.

Pre-processed MRI scans of 40 healthy young adults (20 females and 20 males, mean age of 28 ± 3.9 years), including diffusion-weighted images and a semi-automated FreeSurfer’s atlas, were imported from the Human Connectome Project (HCP) database (Figure 1A) [12]. The multi-shell diffusion scheme was collected on a 3T scanner with a 64-channel, tight-fitting, brain-array coil. The b-values were 1,000, 2,000, and 2,995 s/mm^2^. The diffusion sampling directions were 90, 90, and 90, respectively. The in-plane resolution was 1.25 mm, and the slice thickness was 1.25 mm.

The sample selection process involved randomly selecting participants from the HCP database, prioritizing those with complete and valid diffusion-weighted imaging (DWI) data from 3Tesla MRI scans. Due to the homogeneous nature of the HCP population consisting of healthy young adults, we randomly chose 20 males and 20 females to form a sample size of 40, in line with common practices observed in tractography studies. The decision to adhere to this sample size range is driven by the manual inspection and refinement required for constructed streamlines, which makes larger sample sizes impractical. Notably, prominent studies in the field have similarly utilized sample sizes within this range, reflecting the standard approach in managing the manual workload while ensuring study integrity.

The term 'gender' used in this study aligns with the classification system employed in the HCP dataset, which defines gender based on a binary categorization into groups denoted as 'M' (male) and 'F' (female) according to the HCP data dictionary.

GQI tractography

The diffusion data were reconstructed with a diffusion sampling length ratio of 1.25 [11]. A deterministic fiber-tracking algorithm was also applied [13]. Tractography was conducted using the DSI Studio software tool (https://dsi-studio.labsolver.org), and tractography masks were imported from semi-automated FreeSurfer’s atlas. The normalized quantitative anisotropy threshold was set to 0.075, and the angular threshold was 45 degrees. Streamline trajectories were smoothed by averaging the propagation direction with 20% of the previous direction.

Streamline selection

The tractography procedure for the whole sample is made through the DSI-Studio command line interface. The process of constructing CC streamlines involved utilizing Freesurfer’s atlas. By default, the CC is partitioned into five segments through automatic parcellation: posterior, mid-posterior, central, mid-anterior, and anterior segments. Each segment served as an individual region of interest (ROI) in separate tractography runs. Additionally, the left hemisphere’s white matter was designated as the seed region, while the right hemisphere’s white matter functioned as the end region (Figure 1B). This approach enabled the reconstruction of streamlines starting from the left hemisphere, passing through the selected CC segment, and ending in the right hemisphere. Figure 1 displays an illustration depicting the five constructed segments of CC streamlines within a single subject. Each generated sample underwent visual inspection to ensure error-free processing.

Following this, using DSI-studio’s command line interface, we retrieved FA and volume values for each sample (Figure 1C). The volume values were subsequently standardized relative to the total white matter volume, allowing for the quantification of normalized volumes.

Cortical distribution

The construction of a graphical group overlap image is a technique that can help with group-level inferences about the number of participants in an experiment. This method involves overlaying a group of binary images that can be summed onto a standard anatomical template. This allows visualization of how many subjects showed the structure of interest at each voxel. During the overlapping process, some voxels may have a higher frequency of the CC segments than others. These differences in voxel frequency can be displayed using a color palette in a single image, known as the overlap image.

Consequently, the generated CC segment streamlines underwent conversion into 3D volumetric masks utilizing DSI-Studio’s *Tract to ROI* tool (Figure 1D). Subsequently, these newly formulated masks were registered to the Montreal Neurological Institute (MNI) standard space and subjected to binarization through the utilization of *flirt* and *fslmaths* functions from the FSL package (FSL, University of Oxford, Oxford, UK) (Figure 1E). The creation of overlap images (Figure 1F) was conducted employing the MRIcron software (www.nitrc.org/projects/mricron). The overlap map, derived from the summation of binarized CC representations within each gender group, illustrates the count or total number of occurrences of callosal fibers per voxels. Each voxel value signifies the occurrence count, revealing the spatial prevalence of CC presence across individuals within the respective gender group. 

Additionally, subtraction plots (Figure 1G) were generated utilizing *fslmaths* functions from the FSL package. The subtraction plot presented in this study is generated by subtracting the overlap maps of males from females and portrays the gender-specific spatial differences in callosal voxel occurrences. Positive values highlight areas where the number of occurrences of the corpus callosum within voxels is notably higher in females than in males. Conversely, negative values represent regions where males exhibit higher counts compared to females.

The outcome images were displayed over a two-dimensional (2D) MNI template or a three-dimensional (3D) brain surface average template with the aid of the open-source Workbench Viewer (*wb_view*) software from HCP (Washington University in St. Louis and affiliated consortium institutions; https://www.humanconnectome.org/). The subtraction plots were projected onto a 3D brain surface template in MNI space by utilizing the HCP workbench command line interface software (*wb_command*) through the *-volume-to-surface-mapping* tool.

Our results will exclusively feature the subtraction plot overlaid on 3D brain surface templates, with the 2D overlap and subtraction images omitted.

Statistical analysis

The descriptive statistics were presented as mean ± standard error of the mean (SE). To analyze the differences in FA between the gender groups, Student’s t-test was employed after confirming the assumptions of equal variances and normality.

We investigated gender-based differences in the normalized volume of the CC and its five segments with the white matter volume included as a covariate to control for its potential influence on volume measurements, ensuring that observed differences were specifically attributable to gender and not confounded by variations in white matter volume. Analysis of covariance (ANCOVA) was selected as the primary analytical approach. Prior to conducting the ANCOVA analysis, several assumptions were evaluated to ensure the validity of the results. These included the assessment of normality, evaluation of homogeneity of variances, examination of homogeneity of regression slopes, and verification of linearity.

To account for multiple comparisons, the Benjamini-Hochberg method was employed for p-value adjustment in all previously mentioned tests. Corresponding effect sizes (Cohen’s *d* for Student's t-test and generalized eta squared (*η^2^_G_*) for ANCOVA) were reported alongside the respective adjusted *p*-values. The statistical analyses were conducted using the R statistical package (version 4.3.2; R Development Core Team, Vienna, Austria) and JASP statistical analysis software (version 0.18.2; University of Amsterdam, Amsterdam, Netherlands).

## Results

The FA and normalized volume

Table 1 summarizes the mean and SE of the FA and normalized volume. In the latter, females consistently showed higher mean values compared to males.

**Table 1:** The mean and standard error (SE) of fractional anisotropy and normalized volume.

	Gender	Fractional anisotropy		Normalized volume	
		Mean	SE	Mean	SE
Posterior	F	0.79	5.41×10^-3^	8.48	0.29
	M	0.78	5.95×10^-3^	7.63	0.15
Mid-posterior	F	0.77	3.62×10^-3^	3.76	0.22
	M	0.76	4.27×10^-3^	3.16	0.15
Central	F	0.76	4.68×10^-3^	4.28	0.22
	M	0.75	4.83×10^-3^	3.43	0.16
Mid-anterior	F	0.74	5.98×10^-3^	3.99	0.21
	M	0.73	6.32×10^-3^	3.37	0.11
Anterior	F	0.72	6.71×10^-3^	5.58	0.22
	M	0.71	6.93×10^-3^	5.23	0.19
Total bundle	F	0.75	4.37×10^-3^	26.09	0.93
	M	0.75	4.85×10^-3^	22.8	0.52

**Figure 2:**
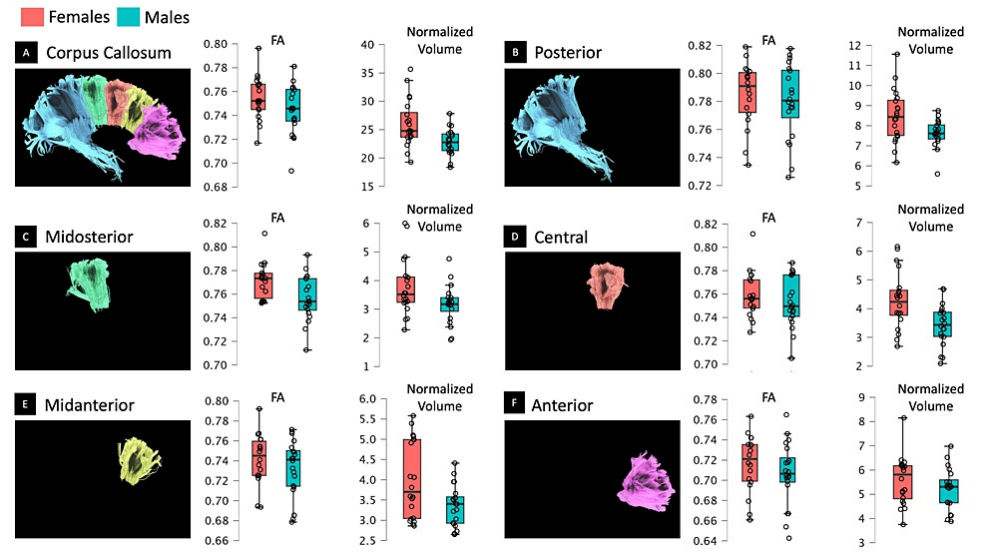
Tractography of the CC and analysis of mean differences. The left images display streamlines of the CC and its five segments. The middle plots feature violin boxplots illustrating the FA mean difference. The right plots depict violin boxplots demonstrating the analysis of the normalized volume mean difference. FA: fractional anisotropy

Two extreme outliers were identified in the FA results and were omitted from the analysis. There were no significant differences in FA between the two gender groups across the entire CC or its individual five segments (Table 2, Figure 2).

**Table 2:** The analyses of the mean difference between the females and males in the fractional anisotropy values.

	t	p	Adjusted *p* (BH)	Mean difference	95% CI for mean difference		Cohen's *d*	95% CI for Cohen's *d*	
					Lower	Upper		Lower	Upper
Posterior	0.61	0.55	0.55	4.91×10^-3^	-0.01	0.02	0.2	-0.44	0.83
Mid-posterior	2.62	0.01	0.06	0.01	3.37×10^-3^	0.03	0.85	0.18	1.51
Central	0.9	0.38	0.46	6.06×10^-3^	-79.4	0.02	0.29	-0.35	0.93
Mid-anterior	1	0.32	0.46	8.78×10^-3^	-92.7	0.03	0.33	-0.32	0.96
Anterior	1.03	0.31	0.46	0.01	-99.6	0.03	0.34	-0.31	0.97
Total bundle	1.36	0.18	0.46	8.92×10^-3^	-47.2	0.02	0.44	-0.21	1.08
Note. Student's t-test									
BH: Benjamini-Hochberg *p*-value adjustment method									

**Figure 3:**
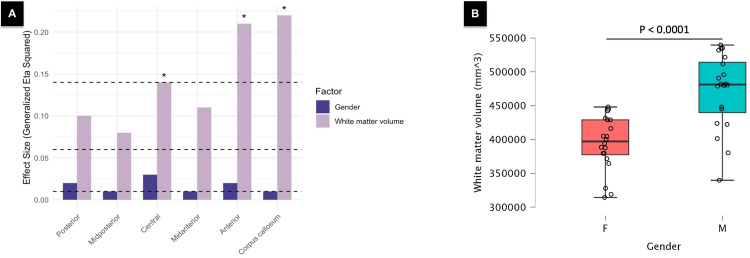
The impact of the total white matter volume on CC and its segments. (A) The bar chart displays* η^2^_G_* values and the distinctions between the effect of gender and total white matter volume factors. The total white matter volume exhibited a large and significant effect (i.e., *η^2^_G_* ≥ 0.14, *p* < 0.05) in analyses of the total CC bundle, central segment, and anterior segment. Horizontal dashed lines indicate thresholds for low (*η^2^_G_* = 0.01), moderate (*η^2^_G_* = 0.06), and large (*η^2^_G_* = 0.14) generalized eta squared (*η^2^_G_*) values. Asterisks denote tests with adjusted *p*-values < 0.05. (B) Box plot illustrating a significant difference in the total white matter volume between males and females (*p* < 0.0001).

Regarding the normalized volume (Table 3), when considering the effects of the factors, we observe varying levels of significance and effect sizes. For the factor 'gender,' there was a lack of significant gender-based differences. In contrast, the 'white matter volume' factor showed significant gender-based differences across the central segment (*F*(1,37) = 6.00, *p* = 0.03, *η^2^_G_* = 0.14), the anterior segment (*F*(1,37) = 9.57 *p* = 0.01, *η^2^_G_* = 0.21), and the total corpus callosum (*F*(1,37) = 10.35, *p* < 0.0001, *η^2^_G_* = 0.22) (Figure 3A).

**Table 3:** The analysis of covariance (ANCOVA). Values in bold font represent results with significant effect.

Test	Effect	SSn	SSd	DFn	DFd	F	Raw *p*	BH-adj *p*	η^2^_G_
Posterior	(Intercept)	57.29	36.74	1	37	57.69	< 0.0001	< 0.0001	0.61
	Gender	0.62	36.74	1	37	0.63	0.43	0.43	0.02
	White matter volume	4.23	36.74	1	37	4.26	0.05	0.07	0.1
Midposterior	(Intercept)	14.77	24.75	1	37	22.07	< 0.0001	< 0.0001	0.37
	Gender	0.28	24.75	1	37	0.42	0.52	0.52	0.01
	White matter volume	2.19	24.75	1	37	3.27	0.08	0.12	0.08
Central	(Intercept)	21.44	24.63	1	37	32.22	< 0.0001	< 0.0001	0.47
	Gender	0.68	24.63	1	37	1.02	0.32	0.32	0.03
	White matter volume	3.99	24.63	1	37	6	0.02	0.03	0.14
Midanterior	(Intercept)	16.73	19.41	1	37	31.88	< 0.0001	< 0.0001	0.46
	Gender	0.28	19.41	1	37	0.53	0.47	0.47	0.01
	White matter volume	2.48	19.41	1	37	4.73	0.04	0.054	0.11
Anterior	(Intercept)	39.57	26.12	1	37	56.05	< 0.0001	< 0.0001	0.6
	Gender	0.61	26.12	1	37	0.87	0.36	0.36	0.02
	White matter volume	6.76	26.12	1	37	9.57	0	0.01	0.21
Corpus callosum	(Intercept)	697.82	336.67	1	37	76.69	< 0.0001	< 0.0001	0.68
	Gender	3.56	336.67	1	37	0.39	0.54	0.54	0.01
	White matter volume	94.13	336.67	1	37	10.35	0	< 0.0001	0.22
SSn: sums of squares for the numerator (effect); SSd: sums of squares for the denominator (error); DFn and DFd represent the degrees of freedom for the numerator and denominator, respectively; F: F-statistic; BH-adj p: Benjamini-Hochberg adjusted p-value; η²G: generalized eta squared									

Hence, it can be inferred that the elevated mean values observed in the normalized volume of the CC among females, particularly in the total CC bundle, the anterior segment, and the central segment, are influenced by differences in the absolute white matter volume. To confirm this observation, we conducted an additional analysis focusing on gender disparities in the white matter volume. Student’s t-test revealed a significantly greater white matter volume in males compared to females (*t*(38) = -5.05, *p* < 0.0001, Cohen’s *d* = -1.6) (Figure 3B, Table 4).

**Table 4:** Independent samples t-test results showing the gender-based difference in the white matter volume.

	t	df	p	Mean difference (F-M)	SE	95% CI for mean		Cohen's *d*	95% CI for Cohen's *d*	
						Lower	Upper		Lower	Upper
White matter volume	-5.05	38	< 0.0001	-77392	15315	-108396	-46388	-1.6	-2.38	-0.786
t: t-statistics; df: degree of freedom; SE: standard error; CI: confidence interval										

Cortical distribution

**Figure 4:**
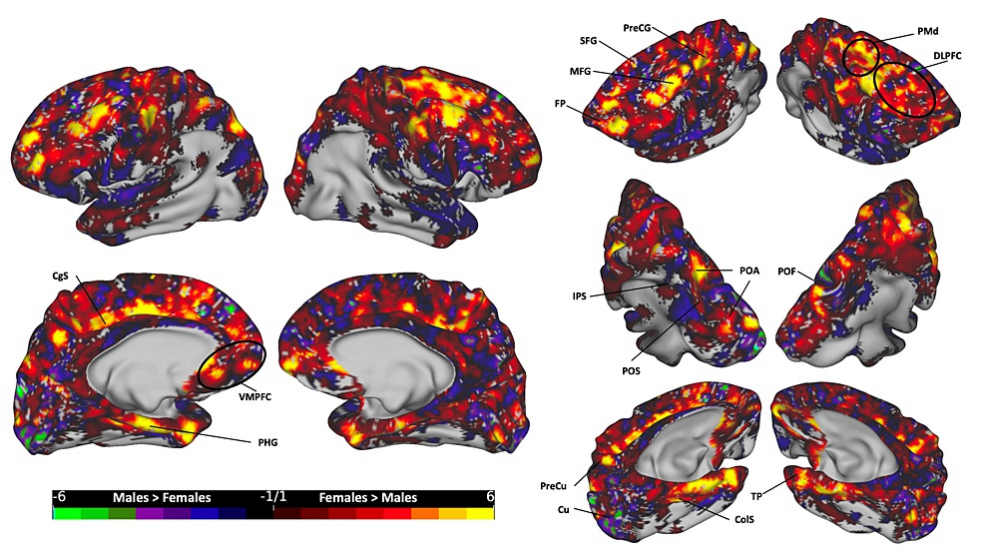
The subtraction plot of the entire CC bundle. The plot is superimposed on a three-dimensional (3D) average brain surface template. The color bar displays warmer hues for a higher occurrence among females, while cooler hues indicate higher male occurrence. CgS: cingulate sulcus; ColS: collateral sulcus; Cu: cuneus; DLPFC: dorsolateral prefrontal cortex; FP: frontal pole; IPS: intraparietal sulcus; MFG: middle frontal gyrus; PHG: parahippocampal gyrus; PMd: dorsal premotor; POA: paroccipital arcus; POF: parieto-occipital fissure; POS: paroccipital sulcus; PreCG: precentral gyrus; PreCU: precuneus; SFG: superior frontal gyrus; TP: temporal pole; VMPFC: ventromedial prefrontal cortex

The subtraction plots of the total CC bundle (Figure 4) revealed higher female occurrence in several cortical regions, including the bilateral superior and middle frontal gyri (SFG and MFG), incorporating the dorsal premotor (PMd), dorsolateral prefrontal cortex (DLPFC), and frontal pole (FP); bilateral motor cortex in the precentral gyrus (PreCG); bilateral parahippocampal gyrus (PHG), collateral sulcus (ColS), and temporal poles (TP); bilateral ventromedial prefrontal cortex (VMPFC); bilateral superior parietal lobule (SPL) and lateral occipital (LO) cortex, particularly in areas lateral to the interparietal sulcus (IPS) and paroccipital sulcus (POS) in the paroccipital arcus (POA), with a stronger presence on the left side; and the right cuneus (Cu), right precuneus (PreCu), and bilateral cingulate sulcus (CgS). Conversely, males exhibited higher rates in the left Cu and right parieto-occipital fissure (POF).

**Figure 5:**
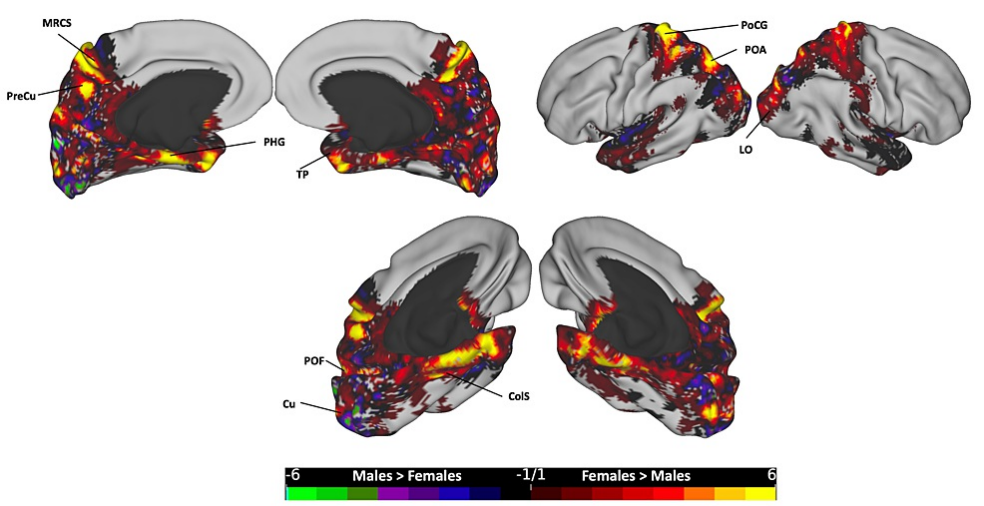
The subtraction plot of the posterior segment of the corpus callosum. The plot is superimposed on a 3D average brain surface template. The color bar displays warmer hues for a higher occurrence among females, while cooler hues indicate higher male occurrence. ColS: collateral sulcus; Cu: cuneus; LO: lateral occipital; MRCS: marginal ramus of the cingulate sulcus; POA: paroccipital arcus; PHG: parahippocampal gyrus; PoCG: postcentral gyrus; POF: parieto-occipital fissure; PreCu: precuneus; TP: temporal pole

In the posterior segment (Figure 5), females exhibited higher occurrence in several areas, including bilateral dorsal postcentral gyrus (PoCG), bilateral lateral SPL, especially in the left POA area, bilateral marginal ramus of the cingulate gyrus (MRCS), left POF, left PreCu, bilateral PHG and TP, bilateral anterior part of the ColS, and bilateral LO cortex. Conversely, males showed more count in the left Cu at the medial occipital area.

**Figure 6:**
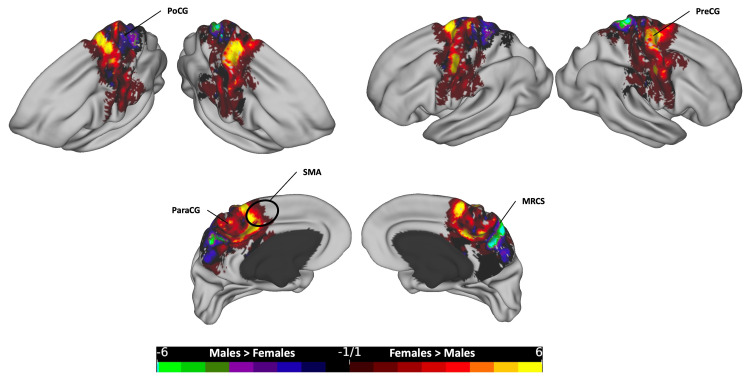
The subtraction plot of the mid-posterior segment of the corpus callosum. The plot is superimposed on a 3D average brain surface template. The color bar displays warmer hues for a higher occurrence among females, while cooler hues indicate higher male occurrence. MRCS: marginal ramus of the cingulate sulcus; ParaCG: paracingulate gyrus; PreCG: precentral gyrus; PoCG: postcentral gyrus; SMA: supplementary motor area

In the mid-posterior segment (Figure 6), females exhibited higher values in the bilateral dorsal PreCG and bilateral paracentral gyrus (ParaCG), extending ventrally to the cingulate cortex and the caudal part of the supplementary motor area (SMA) in the dorsomedial frontal cortex. On the other hand, males demonstrated higher values in the bilateral MRCS area, extending to the dorsal part of the PoCG.

**Figure 7:**
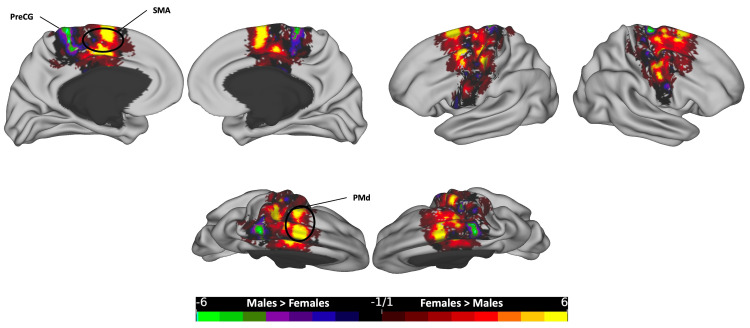
The subtraction plot of the central segment of the corpus callosum. The plot is superimposed on a 3D average brain surface template. The color bar displays warmer hues for a higher occurrence among females, while cooler hues indicate higher male occurrence. PreCG: precentral gyrus; PMd: dorsal premotor; SMA: supplementary motor area

In the central segment (Figure 7), a higher count for females was observed in the bilateral dorsal PreCG, bilateral PMd area, and bilateral SMA in the dorsomedial frontal cortex. On the other hand, males have shown more occurrence in the most dorsal part of the bilateral PreCG extending to the medial surface (lower limb motor area).

**Figure 8:**
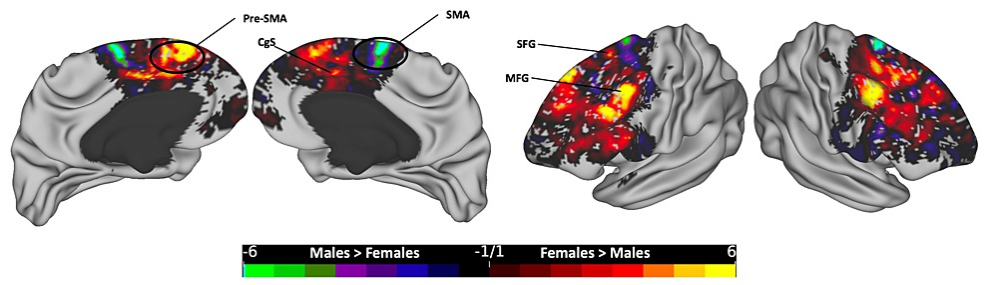
The subtraction plot of the mid-anterior segment of the corpus callosum. The plot is superimposed on a 3D average brain surface template. The color bar displays warmer hues for a higher occurrence among females, while cooler hues indicate higher male occurrence. CgS: cingulate sulcus; MFG: middle frontal gyrus; Pre-SMA: pre-supplementary motor area; SFG: superior frontal gyrus; SMA: supplementary motor area

In the mid-anterior segment (Figure 8), females exhibited higher rates of occurrence in the bilateral MFG, parts of the bilateral superior frontal sulcus (SFS) and SFG, bilateral pre-SMA in the dorsomedial frontal cortex, and bilateral anterior CgS. Conversely, the number of males was higher in the most caudal and dorsal part of the bilateral SFG, extending to the medial surface at the SMA.

**Figure 9:**
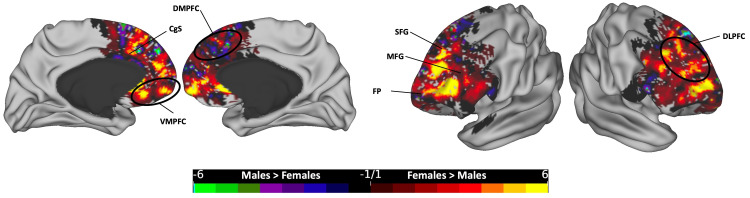
The subtraction plot of the anterior segment of the corpus callosum. The plot is superimposed on a 3D average brain surface template. The color bar displays warmer hues for a higher occurrence among females, while cooler hues indicate higher male occurrence. CgS: cingulate sulcus; DLPFC: dorsolateral prefrontal cortex; DMPFC: dorsomedial prefrontal cortex; FP: frontal pole; MFG: middle frontal gyrus; SFG: superior frontal gyrus; VMPFC: ventromedial prefrontal cortex

In the anterior segment (Figure 9), females predominated in several regions, including the bilateral DLPFC and FP areas; bilateral dorsomedial and ventromedial prefrontal cortices (DMPFC and VMPFC), with a greater prevalence noted in the left hemisphere; and the bilateral anterior part of the CgS, with a higher occurrence on the left hemisphere. Conversely, males occupied more voxels in certain areas of the SFG, extending to the medial surface at the DMPFC.

## Discussion

The current study delves into the gender-based differences regarding the morphology of the tractography-constructed streamlines of the CC. Our assessment of FA values revealed no discernible gender-based differences, a finding conflicting with prior reports. However, it is crucial to note that most prior studies centered on the mid-sagittal CC structure, while our study measured mean FA within the interconnecting commissural streamlines. Inconsistencies exist among these reports, with some indicating higher FA values in females and others in males. For instance, Kanaan et al. reported higher FA in females, specifically within the CC genu segment [7]. Conversely, Oh et al. observed lower regional FA values in females across the entire CC yet significantly higher values in the rostrum, genu, and splenium [14]. In contrast, Liu et al. reported lower FA values in females within the CC genu compared to males, offering contrasting findings from previous studies [15].

In line with previous studies examining midsagittal volume [5,6,8,9], females demonstrated larger normalized CC volumes compared to males (Table 1). However, upon adjusting for mean differences by including the total white matter volume as a covariate in the analysis, the gender factor did not yield a significant effect (Figures 2-3A, Table 3). Conversely, the white matter volume exhibited a notable influence on the gender-based difference of the total CC (*p* < 0.0001, *η^2^_G_* = 0.22), central CC (*p* < 0.03, *η^2^_G_* = 0.14), and anterior CC (*p* < 0.01, *η^2^_G_* = 0.21). Notably, the anterior segment emerged as having a predominant impact on the overall effect observed in the total whole CC (Figure 3A). This phenomenon is predominantly attributed to the significantly larger absolute white matter volume in males (*p* < 0.0001, *d* = 1.6; Figure 3B, Table 4).

The larger effect size observed in the total white matter volume could potentially be attributed to disparities in brain size between genders, possibly influenced by differences in skull size. Several studies have highlighted gender-based variations in the shape and size of specific skull bones, such as the glabella, superciliary arches, and lateral side of the frontal bone, suggesting a structural foundation for observed differences [16-18]. The coordinated growth of the brain and skull during development is well-documented, with the expanding brain exerting pressure on skull bones, prompting them to enlarge to accommodate brain growth [19,20]. This process is most active during infancy and childhood, coinciding with periods of rapid brain development. Given the prominence of frontal bones in males, it is plausible that this anatomical distinction could influence the size of the frontal part of the human brain, subsequently affecting the anterior segment of the CC. Notably, gender-based differences in the frontal white matter size may manifest early in development, as indicated by analyses of MR imaging growth trajectory patterns over 18 gestational weeks. These analyses revealed significant asymmetry in frontal white matter volume between genders, with greater asymmetry observed in males [21].

Following registration to the MNI standardized template, we analyzed the gender-based distribution of streamlines across hemispheres. Significantly, females exhibit higher occurrence rates in cortical regions covered by the total CC streamlines compared to males (Figure 4). Such areas included the bilateral PreCG, DLPFC, PMd, and SPL, as well as the medial temporal cortex, encompassing the PHG, ColS, and temporal poles. Additionally, higher female rates were observed in the CgS and the VMPFC areas. In contrast, regions demonstrating higher rates in males over females were notably limited, primarily localized in the medial occipital region.

Each of the five segments of the CC exhibited distinct cortical distribution patterns, with females consistently showing broader distributions and more frequent occurrences across all segments. In the posterior segment, females surpassed males in the lateral SPL, medial parietal, and medial temporal regions, while males exhibited higher rates in the medial occipital zone, particularly in the left hemisphere (Figure 5). In the mid-posterior segment, females had higher rates in the dorsal part of the primary motor cortex and ParaCG on the medial surface, extending anteriorly to the SMA zone, while males showed dominance in hotspots at the most dorsal part of the bilateral PoCG, extending rostrally to the SPL and medially to the PreCu and MRCS (Figure 6). Moving to the central segment, females exhibited higher rates in the bilateral SMA and PMd areas, whereas males showcased dominance in the most dorsal PreCG region, spanning medially over the lower limb, trunk, and shoulder motor areas (Figure 7). In the mid-anterior and anterior segments, females demonstrated higher rates in the lateral and medial frontal cortex, including the CgS, and males displayed dominance at the medial surface in the SMA area, contrasting with the female dominance observed in the central segment at the same site (Figures 8-9). This variation may elucidate the absence of a distinct hotspot in the SMA for the total CC bundle (Figure 4), indicating diverse connectivity patterns for each CC segment across gender groups.

The comparison between segmented and total bundle analyses of the CC in gender-based distribution revealed nuanced disparities, as we saw in the SMA. While the holistic assessment offered by the total bundle method provides an overall perspective, segmenting the CC unearthed specific cortical hotspots exhibiting gender variation. This implies a gender-dependent density and distribution of connections within CC segments, suggesting a complex interaction between gender and regional CC connectivity. Furthermore, the observation that fibers originating from specific points in one hemisphere may not terminate precisely in corresponding areas in the opposite hemisphere hints at a more intricate distribution of commissural fibers than previously assumed.

Ingalhalikar et al. conducted a diffusion tensor imaging study on healthy young adults, corroborating our findings by demonstrating that females exhibit stronger interhemispheric connections compared to males [22]. Their illustrations of the edges of the connection-wise analysis between the nodes in the hemispheres support our suggestion that connections may not occur strictly between mirror points. Comprehensive investigations incorporating gender-based connectome analyses are warranted to elucidate the neural mechanisms underpinning these segment-specific connectivity disparities and their potential functional implications.

Pinpointing the exact reasons for the different cortical distribution of streamlines between males and females is challenging. Nonetheless, these cortical differences likely echo various cognitive function discrepancies documented between genders. For instance, studies suggest that males often excel in tasks requiring superior motor skills such as aiming, catching, and spatial processing, while females exhibit more refined fine motor skills and enhanced memory functions, especially in episodic memory and facial recognition [23-26]. The gender-based disparities found in our results in the motor and premotor cortex align with this research's findings, highlighting males’ superior motor abilities and females’ proficiency in fine motor skills.

Similarly, the observed variations in memory-related areas, such as the PHG, could signify females’ more efficient access to and precision in memory recall [27,28]. Furthermore, differences in cortical activation within the ColS, implicated in face recognition, mirror those in studies indicating females’ advantage in recognizing faces [29], particularly those of their own gender [30-33].

The current inferences, while insightful, are speculative, highlighting the need for dedicated investigations into both functional and structural connectivity. These explorations aim to unravel the intricate mechanisms underpinning the observed gender-based differences in the cortical distribution of the CC white fiber bundle. While some researchers contend that gender disparities in the brain are not solely from structural variances but are multifaceted, encompassing factors such as socioeconomic status, aging, and educational level [34], recent studies illuminate the impact of social gender inequalities on brain structure. For instance, Zugman et al. demonstrated that women residing in societies with pronounced gender disparities exhibit structural brain alterations potentially linked to poorer mental health and academic attainment [35].

Therefore, the comprehension of brain structural disparities extends beyond innate or genetic determinants, encompassing influences from environmental and socio-cultural factors. Additionally, these disparities are subject to the direct impact of gonadal hormones and sex chromosome effects, presenting a multifaceted interplay of biological, environmental, and genetic components in shaping brain structural differences [1]. This recognition underscores the complexity surrounding brain differences and emphasizes the influence of various external factors that contribute to its structural variance.

What sets the current study apart from previous research is its unique approach to analyzing the CC structure by examining the entirety of commissural fibers rather than solely focusing on the midsagittal structure. This provides novel insights into gender-based studies on brain structure. Additionally, the meticulous analysis of covariates revealed that gender-based differences in the CC are significantly influenced by the absolute white matter volume. This highlights the need to broaden the scope of research in this field to encompass additional factors beyond gender. Future investigations should aim to elucidate the underlying reasons for the pronouncedly larger frontal segments of the CC in males compared to females. Such phenomena may be driven by genetic or hormonal factors that warrant further exploration.

This study has limitations. Although the sample selection process was randomized, no effort was made to align the intracranial volumes of both gender groups. However, for the analysis of post-tractography streamlines' volume or FA values, such alignment is not considered mandatory. The practice of measuring the volume ratio to the entire white matter compensates for these differences and is commonly employed in tractography studies [4,36-39]. Another limitation pertains to the mapping of group-average volume data onto surfaces, such as with the subtraction plots. These methods encounter difficulties in consistently aligning cortical ribbon contours and may diminish folding detail in average surfaces [40].

Additionally, it is important to acknowledge the limitations of the relatively modest sample size comprising 20 males and 20 females. There are various advantages observed in investigations of sex differences in neuroanatomy using large datasets [1]. However, it is pertinent to note that the limited sample size in our study was primarily due to computational constraints. Conducting analyses, particularly in the realm of tractography with extensive datasets, necessitates substantial computational power and memory resources that are often unavailable to all researchers. Consequently, while the findings presented herein shed light on gender-based disparities in the cortical distribution of brain structures, the study's limited sample size might impede the robustness and generalizability of the observed effects. Validation and further exploration using more extensive cohorts are warranted to corroborate and expand upon these findings, leveraging larger-scale studies to enhance statistical power and broaden the applicability of the results.

Lastly, despite advancements in fiber-tractography techniques, a consensus on the precise anatomy of white matter tracts in the human brain remains elusive, lacking a universally accepted framework for defining a tract with specific descriptors that detail its shape and the convergence locations of axons within the deep white matter [41]. Moreover, the impact of variables such as tractography seeding strategy, step size, angular threshold, or interpolation method on the efficacy of tracking algorithms in determining the volume of streamlines and the mean FA cannot be disregarded [13]. Given the absence of standardization in tractography procedures, it is common to encounter varied findings in research reports, as each scientist tends to favor their preferred approach [4].

## Conclusions

Utilizing tractography methods, we examined the morphology of CC fibers and observed no notable fractional anisotropy variations between genders. However, we consistently identified differences in normalized CC volumes, but these differences were predominantly influenced by the effect of the absolute white matter volume, rather than gender-specific effects. The study also revealed higher female cortical distribution dominance in various cortical regions, contrasting with males in specific areas.

These findings emphasize the complexity of gender-based connectivity disparities and their potential implications for cognitive function. Further research is needed to elucidate the multifaceted interplay of biological, environmental, and socio-cultural factors shaping brain structural differences between genders.
